# Optimising age adjustment of trichiasis prevalence estimates using data from 162 standardised surveys from seven regions of Ethiopia

**DOI:** 10.1080/09286586.2018.1555262

**Published:** 2018-12-28

**Authors:** Colin K. Macleod, Travis C. Porco, Michael Dejene, Oumer Shafi, Biruck Kebede, Nebiyu Negussu, Berhanu Bero, Sadik Taju, Yilikal Adamu, Kassahun Negash, Tesfaye Haileselassie, John Riang, Ahmed Badei, Ana Bakhtiari, Rebecca Willis, Robin L. Bailey, Anthony W. Solomon

**Affiliations:** a Clinical Research Department, London School of Hygiene & Tropical Medicine, London, UK; b Francis I. Proctor Foundation, Department of Ophthalmology, University of California, San Francisco, San Francisco, USA; c Department of Epidemiology and Biostatistics, University of California, San Francisco, San Francisco, USA; d Michael Dejene Public Health Consultancy Services, Addis Ababa, Ethiopia; e Federal Ministry of Health, Addis Ababa, Ethiopia; f The Fred Hollows Foundation Ethiopia, Addis Ababa, Ethiopia; g Department of Ophthalmology, Addis Ababa University, Addis Ababa, Ethiopia; h AMREF Health Africa Ethiopia, Addis Ababa, Ethiopia; i ORBIS International, Addis Ababa, Ethiopia; j Gambella Regional Health Bureau, Gambella, Ethiopia; k Department of Disease Prevention, Somali Regional State Health Bureau, Jigjiga, Ethiopia; l Task Force for Global Health, Decatur, Georgia, USA; m Department of Control of Neglected Tropical Diseases, World Health Organization, Geneva, Switzerland

**Keywords:** Trachoma, trichiasis, population-based prevalence survey, Global Trachoma Mapping Project

## Abstract

**Purpose**: The prevalence of trichiasis is higher in females and increases markedly with age. Surveys carried out in the daytime, particularly in developing countries, are prone to find older individuals and females at home at the time of the survey. Population-level trichiasis estimates should adjust sample proportions to reflect the demographic breakdown of the population, although the most accurate method of doing this is unclear.

**Methods**: Having obtained data from 162 surveys carried out in Ethiopia as part of the Global Trachoma Mapping Project from 2012 to 2015, we used internal validation with both Brier and Logarithmic forecast scoring to test stratification models to identify those models with the highest predictive accuracy. Selection of partitions was undertaken by both simple random sampling (SRS) and cluster sampling (CS) over 8192 selections.

**Results**: A total of 4529 (1.9%) cases of trichiasis were identified from 241,139 individuals aged ≥15 years from a total of 4210 kebeles and 122,090 households visited. Overall, the binning method using 5-year bands from age 15 to 69 years, with coarser binning in 20-year age-bands above this age, provided the best predictive accuracy, in both SRS and CS methodologies and for both the Brier and Logarithmic scoring rules.

**Conclusion**: The greatest predictive accuracy for trichiasis estimates was found by adjusting for sex and in 5-year age-bands from the age of 15 to 69 years and in 20-year age-bands in those aged 70 years and greater. Trichiasis surveys attempting to make population-level inferences should use this method to optimise surgery backlog estimates.

## Introduction

Trachoma is an eye disease caused by infection with the bacterium *Chlamydia trachomatis*. It is thought to account for the blindness or visual impairment of 1.9 million people worldwide.^1^ Repeated conjunctival infections^2^ contribute to trichiasis,^3^ associated with chronic, painful irritation of the cornea leading to permanent opacification, visual impairment, and blindness.^4^ Surgery to correct trichiasis is the recommended means by which further vision loss can be minimised, and in many countries, ophthalmic nurses are trained to carry out these corrective surgeries. However, training trichiasis surgeons is costly, and the numbers required are largely informed by the estimated number of eyes requiring surgery in a given area. Accurate population-level estimates of the trichiasis prevalence help guide planning for recruitment and training and enable confident assessment of whether or not districts have reached the World Health Organization (WHO) elimination prevalence threshold.

In population-based surveys, the distribution of age and gender in sampled individuals only imperfectly represents the true demographics of the underlying population. This may be due to random variations in the demographics of those sampled or a bias related to the sampling methodology. In trachoma, both female gender and increasing age are strongly associated with trichiasis,^5–10^ and therefore, sample-based estimates of population-level outcomes risk being biased if particular age and gender groups are disproportionately represented in the sample without estimates being weighted accordingly. Disproportionate representation in samples collected via house-to-house surveys is likely because of age- and gender-determined patterns in absence from the home for work and education.

The optimal method for weighting prevalence estimates is unclear. Although gender is generally dichotomised (feminine/female, masculine/male), the ideal way to subdivide the range of ages in a sample is less obvious. At one extreme, we could subdivide individuals by their age in years, but this might lose the underlying variation in age-dependent risk by introducing imprecision in each age-specific estimate. At the other extreme, all individuals could be encompassed by a division that covers the entire range of ages in the sample, but this ignores changes in risk with age. The width of divisions used can be considered as the bandwidth of the underlying function. Cross-validation has been used in cases where bandwidth of a density function needs to be optimised to obtain a smoothed estimate of the underlying function.^11–14^

In this study, we apply a method of cross-validation to examine data from 162 standardised population-based trachoma prevalence surveys carried out in Ethiopia from 2012 to 2015, as part of the Global Trachoma Mapping Project (GTMP).^15^ The accuracy of each age-binning strategy was evaluated using both a Brier score^16^ and a Logarithmic scoring rule.^17^ In addition, we examine potential effects of terminal digit preference in age reporting.

## Methods

All GTMP surveys followed a standardised methodology, the full details of which are described elsewhere.^18,19^ Briefly, in each survey, a two-stage cluster sampling methodology was used, with kebeles, the lowest administrative unit with available population estimates, sampled at the first stage, using a probability proportional to population size approach. At the second stage, a subsection of the kebele known variously as a got, gere, developmental unit, or developmental team was randomly selected on the day of survey, and all residents aged 1 year and over in households within this selected second-stage cluster were eligible for inclusion in the survey.

Local health-care workers were trained to grade trachoma using the WHO simplified trachoma grading system^3,4^ and were required to pass a standardised training course and field-based examination against a certified grader trainer in order to qualify as a grader for the survey. Candidates were assessed on their ability to grade the ocular clinical sign trachomatous inflammation—follicular (TF). Examination results were called to data recorders and recorded in a smartphone application developed for the project. Data were uploaded directly from the smartphones to a secure cloud-based server.

### Models

#### Resolution

Census populations are most commonly presented by gender and age, with a minimum resolution of 5 years. For this reason, credible methods of aggregating age into bins could only have bandwidths in multiples of 5 years. However, we used a 1-year age bin as a control for the finest possible resolution, estimating the populations in these bins as the population of the respective 5-year bin divided by 5.

#### Transition points

In contrast to developed countries, the population of developing countries typically decreases sharply above the age of average life expectancy. This means that at ages greater than the average life expectancy, the number of survey-sampled individuals in any bin decreases, and the data become more sparse. Bin proportion estimates can, therefore, lack precision. With this in mind, it is reasonable to consider larger bin sizes at higher ages to produce more accurate estimate of the true proportion of individuals with trichiasis in these bins. We included a transition from fine to coarse resolution in this analysis, varying the age at which coarser-resolution binning was introduced between 40, 60, 65, and 70 years.

A total of 17 binning models were evaluated that could credibly be used for age adjustment in population-based surveys (Table 1).

**Table 1.** The predictive accuracy of cross-validated age-binning methods in estimating the true prevalence of trichiasis in those aged ≥15 years. Division by gender was included a priori, with variations in the method of age binning considered which could be used in trachoma prevalence surveys. For each sampling method (simple random sampling [SRS] and cluster random sampling [CRS]) and for outcome score (Brier and Logarithmic), the ordered rank of accuracy for each binning method is shown (rank 1 – most accurate, rank 17 – least accurate).

Binning type				Simple random sampling^a^				Cluster random sampling^b^			
Model	1st increment	Transition age	2nd increment	Brier score	Rank^c^	Log score	Rank^c^	Brier score	Rank^c^	Log score	Rank^c^
**A**	**5**	**70**	**20**	0.0178	1	−0.0816	1	0.0178	1	−0.0813	1
***B***	***5***	***70***	***10***	0.0178	2	−0.0816	2	0.0178	2	−0.0813	2
***C***	***5***	***60***	***10***	0.0179	3	−0.0816	5	0.0178	3	−0.0813	4
**D**	***5***	***65***	***20***	0.0179	4	−0.0816	4	0.0178	4	−0.0813	5
***E***	***5***	***65***	***10***	0.0179	5	−0.0816	3	0.0178	5	−0.0813	3
***F***	***5***	***–***	***–***	0.0179	6	−0.0817	6	0.0178	6	−0.0813	6
**G**	***5***	***40***	***10***	0.0179	7	−0.0817	8	0.0178	7	−0.0814	7
***H***	***5***	***60***	***20***	0.0179	8	−0.0817	9	0.0178	8	−0.0814	8
***I***	***10***	***65***	***20***	0.0179	9	−0.0819	13	0.0178	9	−0.0816	11
**J**	***10***	***–***	***–***	0.0179	10	−0.0819	12	0.0178	10	−0.0816	10
***K***	***5***	***70***	***–***	0.0179	11	−0.0817	7	0.0179	14	−0.0816	12
***L***	***5***	***75***	***–***	0.0179	12	−0.0817	10	0.0179	15	−0.0816	13
**M**	**1**	**–**	**–**	0.0179	13	−0.0819	11	0.0178	11	−0.0815	9
***N***	***5***	***40***	***20***	0.0179	14	−0.082	14	0.0178	12	−0.0817	14
***O***	***20***	***–***	***–***	0.0179	15	−0.0828	15	0.0178	13	−0.0825	15
**P**	***50***	***–***	***–***	0.0181	16	−0.088	16	0.0181	16	−0.0877	16
Base rate^d^	***–***	***–***	***–***	0.0184	17	−0.0915	17	0.0184	17	−0.0914	17

^a^Simple random sampling – data partitioned into 95% training/5% test data by randomly sampling individuals.

^b^Cluster random sampling – data partitioned by stratifying the data-set by survey and then selecting one cluster in each. All individuals in this cluster comprised the test data, with individuals in all other clusters comprising the training data.

^c^Ranked accuracy (1 most accurate, 17 least accurate) of binning method by Brier score (lowest score most accurate) and Logarithmic score (lowest absolute value score most accurate). Scores presented have been truncated to four decimal places for brevity; full precision was used for ranks.

^d^Equivalent to probability of trichiasis being constant for all individuals aged ≥15 years of a given gender, with the probability equal to the overall proportion of the ≥15-year-old population of that gender that had trichiasis: (total trichiasis cases [male or female]/total individuals [male or female] examined).

#### Sampling strategies

The accuracy of models was evaluated by internal cross-validation, partitioning the data into training (95%) and test (5%) data-sets. Two methods of partitioning were compared: simple random sampling (SRS) with partitioning at individual level and cluster random sampling (CRS), respecting the clustered design of the survey. In the SRS methodology, individuals were selected by random number generation in the range [0, 1]. In the CRS methodology, all surveys were stratified, and one cluster from each survey was selected randomly. All inhabitants in the selected cluster for each survey were chosen to be the test group, and all participants living in the other clusters in the survey were selected as the training set, with bin-specific proportions derived from a given survey applied to the training set from that survey.

At each iteration, and for each binning type, age- and gender-specific proportions were calculated using the training data-set. These probabilities were then assigned to individuals in the test data-set based on their respective age and gender. In this way, the accuracy of probabilities derived from each strategy could be evaluated by their ability to predict outcomes in the test data-set. The goodness of fit was evaluated by two metrics: a Brier score and a Logarithmic score. The algorithm for partitioning and scoring is shown in Table 2.

**Table 2.** Algorithm for evaluating alternative age bandwidths to optimise the predictive accuracy of trichiasis estimates.

1. For each iteration:		
	i	Partition the data into a training (95%) and test (5%) set using either SRS or CRS.
	ii	Apply each binning method to individuals in the training set and calculate the proportion of individuals with trichiasis in each bin for each method.
	iii	Apply the bin proportions to the equivalent bins applied to the test data-set and compute the Brier score, B, and Logarithmic score, L, via $B\left(r,q\right)=\frac{1}{N}\overset{N}{\underset{j=1}{\sum }}{\left(T_{j}-p_{j}\right)}^{2}$ $L\left(r,q\right)=\frac{1}{N}\;\overset{N}{\underset{j=1}{\sum }}\left[T_{j}\log_{10}p_{j}+\left(1-T_{j}\right)\log\left(1-p_{j}\right)\right]$ where r is the training set, q is the binning method, $T_{j}$ is the vector of the trichiasis results of the individuals in the training data-set, and $p_{j}$ is the probability of trichiasis for a given individual and binning method.
2. Calculate the mean $B\left(q\right)$ and $L\left(q\right)$ over all 8192 iterations.		

SRS: simple random sampling; CRS: cluster random sampling.

In the training data-set, zero-count bins were assigned an artificial probability using a Laplace estimator, with the bin probability assigned as $1/\left(1+N\right)$, where N is the total number of individuals examined in the bin. Similarly, full (100% prevalence) bins were assigned a probability of $1-1/\left(1+N\right)$.

### Test set scores

#### Brier score

For each model, the squared difference between the predicted probability of trichiasis in the $j^{th}$ individual, $p_{j}$, and the proportion of trichiasis in the actual survey findings, $T_{j}$ (0 or 1: the absence or presence of trichiasis), was calculated for all individuals. The mean score for all individuals was calculated for each iteration, expressed as the equation:

$$B\left(r,q\right)=\frac{1}{N}\overset{N}{\underset{j=1}{\sum }}{\left(T_{j}-p_{j}\right)}^{2}$$

with the highest predictive accuracy achieved by minimising this score.

#### Logarithmic score

The Logarithmic score was similarly calculated by assigning a score to each individual. If trichiasis was present, the score was assigned as the logarithm (base 10) of the probability of trichiasis. If trichiasis was absent, the score was assigned as the logarithm (base 10) of 1 − (probability of trichiasis). The Logarithmic score for each model was assigned as the mean of all such values for each individual at each iteration, expressed as the equation:

$$L\left(r,q\right)=\frac{1}{N}\;\overset{N}{\underset{j=1}{\sum }}\left[T_{j}\log_{10}P_{j}+\left(1-T_{j}\right)\log\left(1-P_{j}\right)\right]$$

with the highest predictive accuracy achieved by maximising this score.

The 95% confidence interval for each score was estimated directly by calculating the 2.5th and 97.5th centiles from the ordered list of all score results over all iterations.

## Errors in age reporting

When self-reporting age, it is common for individuals to have biases towards particular figures, known as terminal digit preference.^20^ This usually means that there is an excess of terminal digit 0s and/or 5s in the reported ages of the sampled population. To investigate any effect of errors in self-reporting of age from survey participants, we applied a normal distribution $N\left(\sigma ,p\right)$ around the reported age, considering positively biased (tendency to over-estimate), negatively biased (tendency to under-estimate), and neutral (unbiased uncertainty in an individual’s reported age) distributions. In the positively biased case, the mean was +2 years; in the negatively biased case, the mean was −2 years; and in the neutral case, the mean was 0 year. In each case, a standard deviation, σ, of 2 years was used. Models were applied to data sampled over 8192 iterations.

As a case example, we apply this age distribution to the census data from the largest region in Ethiopia, Oromia, to estimate the error in the backlog of trichiasis that would be associated with each error (neutral bias, positive bias, and negative bias) and report the results as the percentage change in the estimate of the backlog when applying the optimal binning method to this population. As a control, we included the trichiasis backlog estimate that would arise from a constant trichiasis base rate applied to all individuals, irrespective of age.

## Ethical clearance

The overall GTMP protocol and the subsequent protocol to carry out this secondary analysis was approved by the ethics committee of the London School of Hygiene & Tropical Medicine (LSHTM; Ref 6319 and 8355 and 14519). The survey protocol was approved by the ethics committee of each participating regional state in Ethiopia. The World Health Organization Ethics Review Committee exempted this secondary analysis of anonymised data from full review (0002998).

## Results

**Figure 1.**
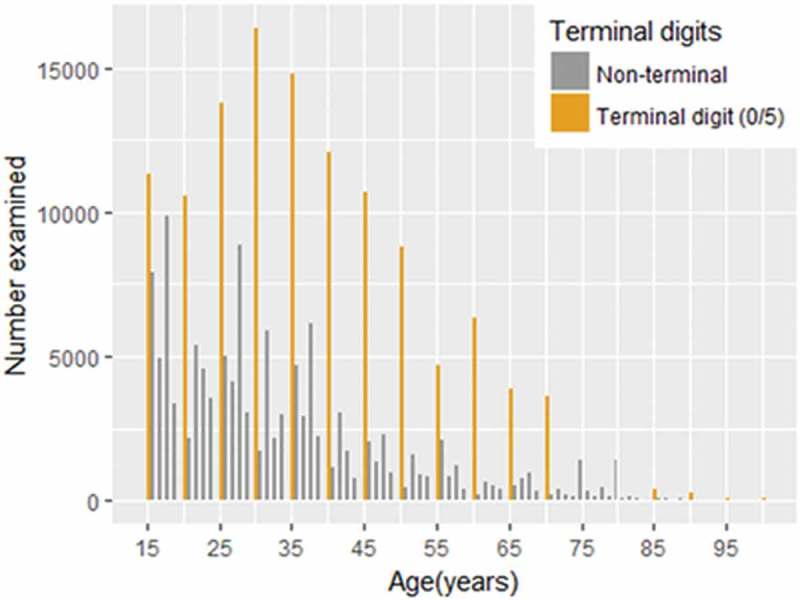
Self-reported ages of ≥15-year-olds examined for trachoma in 162 standardised surveys in seven regions of Ethiopia. In total, 120,656 (50.0%) of 241,137 people examined reported ages with a terminal digit of 0 or 5. Global Trachoma Mapping Project, Ethiopia, 2012–2015.

A total of 241,139 individuals aged ≥15 years were examined in seven regions in Ethiopia in 162 surveys carried out from December 2012 to May 2015. All surveys followed the same two-stage sampling methodology, with a total of 4210 kebeles and 122,090 households visited. Primary analyses are presented elsewhere.^5,6,21–24^ In total, 140,115 (58.1%) of ≥15-year-olds examined were female. Half of all those examined aged ≥15 years reported their age with a terminal digit of 0 or 5 (Figure 1). The median reported age of ≥15-year-olds examined was 32 years (interquartile range [IQR] 24–45; minimum 15, maximum 100). A total of 4529 cases of trichiasis (1.9% of ≥15-year-olds examined) were identified, with 3568 (78.8%) of cases found in females. The median age of those found to have trichiasis was 50 years (IQR 40–64, minimum 15, maximum 100).

The Brier and Logarithmic scores for each binning method, considering SRS and CRS methodologies, as well as the ranked outcomes for each method, are shown in Table 1. Overall, model A, the binning method using 5-year bands with a transition to coarser binning at age 70 years, with 20-year band size above this age, provided the best predictive accuracy in both the SRS and CRS methodologies, and for both the Brier and Logarithmic score outcomes, over 8192 iterations. However, there was near-complete overlap of the 95% confidence intervals (CIs) for models A–H.

**Figure 2.**
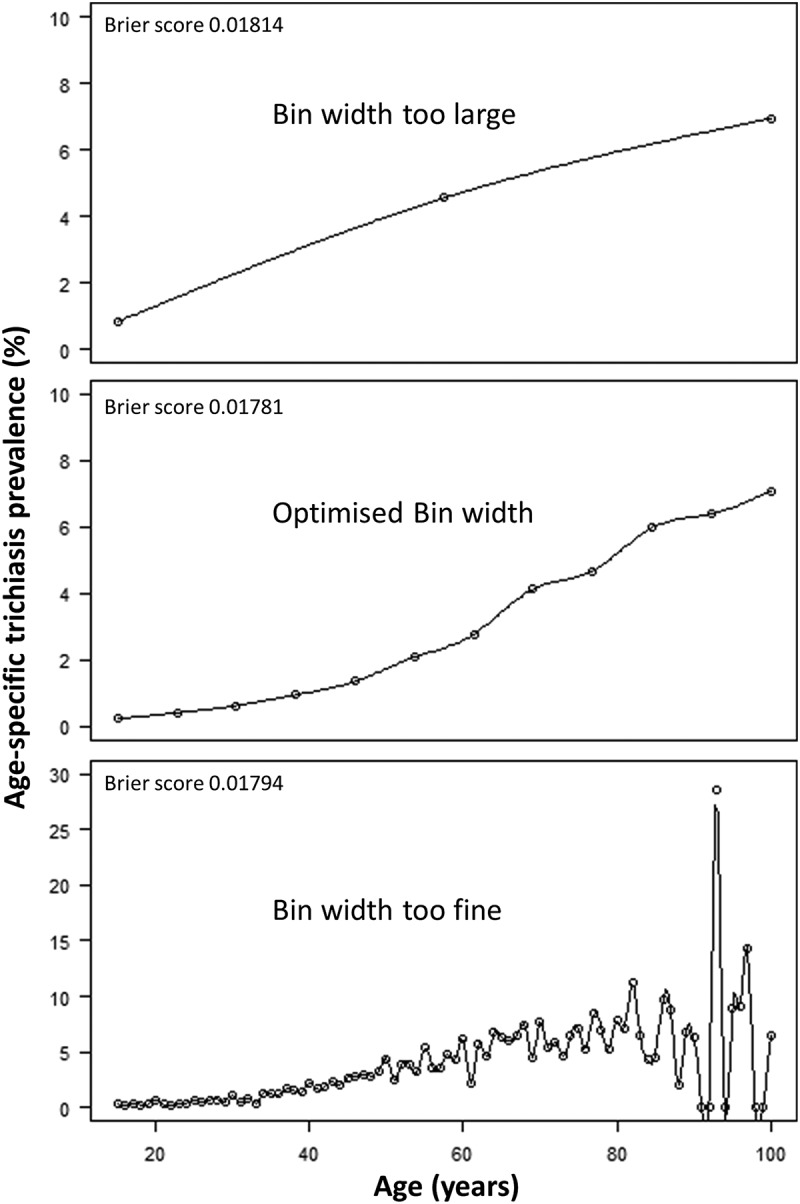
Optimised bin width estimation for maximising predictive accuracy in the age-specific prevalence to trichiasis in those aged ≥15 years using data from 162 standardised surveys carried out in seven regions of Ethiopia. Top: resolution too coarse – 30-year bin widths. Middle: optimised bin width – 5-year binning to age 69 years and then 20-year bins above this age. Bottom: resolution too fine – 1-year bin widths (raw data). Further division by gender not illustrated for simplicity. Global Trachoma Mapping Project, Ethiopia, 2012–2015.

Figure 2 shows the result of the cross-validation method used to optimise the binning choice selection, using model A, the optimal binning method.

## Application of age-reporting bias to trichiasis estimates

We examined the effect of applying the optimised binning method to real-world data, when age variation is allowed in the model. Using the optimised binning method, model A, the adjusted mean trichiasis prevalence in those aged 15 years or greater in Oromia was 1.54%. The mean estimate of the number of trichiasis cases requiring surgery was estimated by applying this figure to the age and gender-specific population estimates from the latest available census data.^25^

The trichiasis backlog estimate from the neutral age distribution had a median value of 228,000 cases (95% CI 226,900–229,000); the backlog from the positive-bias estimate was 206,700 (95% CI 205,800–207,500); the backlog from the negative-bias estimate was 250,000 (95% CI 248,700–251,500; Table 3).

**Table 3.** Estimates of the backlog of cases of trichiasis when applying age distributions to self-reported ages, Global Trachoma Mapping Project, Oromia, Ethiopia.

Age distribution	*Negative bias*	*Neutral*	*Positive bias*
Trichiasis estimate (1000s; 95% CI)^b^	250.0 (248.7–251.5)	228.0 (226.9–229.0)	206.7 (205.8–207.5)

^a^Normal distribution added to reported age with mean 0 (neutral bias), mean +2 (positive bias), and mean −2 (negative bias), and standard deviation 2 years.

^b^Oromia-region estimate of cases of trichiasis; 95% confidence intervals (CIs) from 2.5th and 97.5th centiles of 8192 iterations of model varying age only.

## Discussion

Using a large real-world data-set, we developed a method of cross-validation for optimising bin-size choice for gender- and age-specific trichiasis prevalence standardisation. We evaluated the predictive accuracy achieved using Brier and Logarithmic scores. Using the optimised binning type, we included age variation in subsequent models, accounting for the possibility of inaccuracy in self-reported estimates of age from examined individuals.

The binning method with the highest predictive accuracy divided the population by gender and 5-year-binned age-bands up to 69 years of age and 20-year age-bands above that point. The CIs around the score estimates for each binning type were generally large. However, while we demonstrate that a wide range of binning methods could give comparable predictive accuracy (models A–H), we also demonstrated a range of methods whose bins were either too wide or too narrow and should not be used (models I–Q).

Compared to the neutral-bias estimate, the estimate for the trichiasis backlog was higher in those with a negative-bias in self-reported age (tending to be older than reported). The estimate for the trichiasis backlog was lower in those with a positive bias in self-reported age (tending to be younger than reported). This is intuitively correct, because if a person reports being older than they are, our age-specific trichiasis prevalence (for each gender) will underestimate the true trichiasis prevalence for this age (if the age-reporting bias is not associated with trichiasis status). It is also epidemiologically important, because the prevalence of trichiasis increases with age, with the peak in absolute number of cases at (or near to) the peak in the distribution of ages of individuals whose data contributed to the census. Negatively biasing an age estimate shifts the prevalence curve to the left, so that a higher age-specific trichiasis prevalence is applied to a smaller number of people at population level.

To model the effect of bias in age reporting, we added an age-bias(negative bias, neutral bias, positive bias) from a normal distribution (mean −2, 0, or +2; standard deviation 2) to the self-reported ages. This was chosen pragmatically: we are unable to estimate the degree to which people will provide incorrect information about their true age. The significance of the estimates from the negative-bias and positive-bias models would be dependent on specific cultural knowledge about the tendency to favour higher or lower age estimates. It is possible to conceive of situations where individuals feel they could benefit from either under-estimating or over-estimating their age, and in this sense, age reporting is almost certainly biased to some extent in any survey. However, in our data, this introduced an error around population-level estimates that equated to only ±0.5% of the absolute trichiasis backlog, and this is unlikely to significantly alter planning for surgical services.

It is possible that estimates could be significantly affected if sociocultural factors bias age reporting to a much greater extent than has been considered here, and public health professionals should be aware of this issue. Where this phenomenon is neglected, there is a potential to provide estimates to local planners that may not reflect the reality on the ground, which may result in misallocation of limited resources for trachoma elimination.

## References

[1] Bourne RRA, Stevens GA, White RA, et al Causes of vision loss worldwide, 1990–2010: a systematic analysis. *Lancet Global Health*. 2013;1(6):e339–49. doi:10.1016/S2214-109X(13)70113-X. 25104599

[2] Gambhir M, Basáñez M-G, Burton MJ, et al The development of an age-structured model for trachoma transmission dynamics, pathogenesis and control. *PLoS Negl Trop Dis*. 2009;3(6). doi:10.1371/journal.pntd.0000462. PMC2691478 19529762

[3] Thylefors B, Dawson CR, Jones BR, West SK, Taylor HR. A simple system for the assessment of trachoma and its complications. *Bull World Health Organ*. 1987;65:477–483. 3500800 PMC2491032

[4] Mabey DCW, Solomon AW, Foster A Trachoma. *Lancet*. 2003;362(9379):223–229. doi:10.1016/S0140-6736(03)13914-1. 12885486

[5] Bero B, Macleod C, Alemayehu W, et al Prevalence of and risk factors for Trachoma in Oromia regional state of Ethiopia: results of 79 population-based prevalence surveys conducted with the global trachoma mapping project. *Ophthalmic Epidemiol*. 2016;23(6):392–405. doi:10.1080/09286586.2016.1243717. 27820657 PMC6837860

[6] Adera TH, Macleod C, Endriyas M, et al Prevalence of and risk factors for Trachoma in Southern Nations, Nationalities, and peoples’ region, Ethiopia: results of 40 population-based prevalence surveys carried out with the global trachoma mapping project. *Ophthalmic Epidemiol*. 2016;23(Suppl. 1):84–93. doi:10.1080/09286586.2016.1247876. 27918229 PMC5706981

[7] Smith JL, Sivasubramaniam S, Rabiu MM, Kyari F, Solomon AW, Gilbert C Multilevel analysis of trachomatous trichiasis and corneal opacity in Nigeria: the role of environmental and climatic risk factors on the distribution of disease. *PLoS Negl Trop Dis*. 2015;9(7). doi:10.1371/journal.pntd.0003826. PMC4519340 26222549

[8] Cromwell EA, Courtright P, King JD, Rotondo LA, Ngondi J, Emerson PM The excess burden of trachomatous trichiasis in women: a systematic review and meta-analysis. *Trans R Soc Trop Med Hyg*. 2009;103(10):985–992. doi:10.1016/j.trstmh.2009.03.012. 19362326

[9] Hu VH, Harding-Esch EM, Burton MJ, Bailey RL, Kadimpeul J, Mabey DCW Epidemiology and control of trachoma: systematic review. *Trop Med Int Health*. 2010;15(6):673–691. doi:10.1111/j.1365-3156.2010.02521.x. 20374566 PMC3770928

[10] Elshafie BE, Osman KH, Macleod C, et al The epidemiology of Trachoma in Darfur States and Khartoum State, Sudan: results of 32 population-based prevalence surveys. *Ophthalmic Epidemiol*. 2016;23(6):381–391. doi:10.1080/09286586.2016.1243718. 27841721 PMC5297557

[11] Rudemo M Empirical choice of histograms and kernel density estimators. *Scand J Stat*. 1982;9(2):65–78. http://www.jstor.org/stable/4615859.

[12] Bowman AW An alternative method of cross-validation for the smoothing of density estimates. *Biometrika*. 1984;71(2):353–360. doi:10.2307/2336252.

[13] Loader CR Bandwidth selection: classical or plug-in? Lucent technologies. *Ann Stat*. 1999;27(2):415–438. http://citeseerx.ist.psu.edu/viewdoc/download?doi=10.1.1.462.951&rep=rep1&type=pdf. Accessed 412, 2017

[14] Jones MC, Marron JS, Sheather SJ A brief survey of bandwidth selection for density estimation. *J Am Stat Assoc*. 1996;91(433):401–407. doi:10.2307/2291420.

[15] Solomon AW, Kurylo E The global trachoma mapping project. *Community Eye Health*. 2014;27:18. 24966461 PMC4069783

[16] Brier G Verification of forecasts expressed in terms of probability. *Mon Weather Rev*. 1950;78:1–3. doi:10.1175/1520-0493(1950)078<0001:VOFEIT>2.0.CO;2.

[17] Good IJ Rational decisions. *J R Stat Soc Ser B*. 1952;14(1):107–114. http://www.jstor.org/stable/2984087.

[18] Solomon AW, Pavluck A, Courtright P, et al The global trachoma mapping project: methodology of a 34-country population-based study. *Ophthalmic Epidemiol*. 2015;22(3):214–225. doi:10.3109/09286586.2015.1037401. 26158580 PMC4687001

[19] Solomon AW, Willis R, Pavluck AL, et al Quality assurance and quality control in the global trachoma mapping project. *Am J Trop Med Hyg*. 2018;99(4):858–863. doi:10.4269/ajtmh.18-0082. 30039782 PMC6159583

[20] Denic S, Saadi H, Khatib F Quality of age data in patients from developing countries. *J Public Health (Bangkok)*. 2004;26(2):168–171. doi:10.1093/pubmed/fdh131. 15284321

[21] Adamu Y, Macleod C, Adamu L, et al Prevalence of Trachoma in Benishangul Gumuz Region, Ethiopia: results of seven population-based surveys from the global trachoma mapping project. *Ophthalmic Epidemiol*. 2016;23(sup1):70–76. doi:10.1080/09286586.2016.1247877. 27918248 PMC5706978

[22] Sherief ST, Macleod C, Gigar G, et al The prevalence of Trachoma in Tigray Region, Northern Ethiopia: results of 11 population-based prevalence surveys completed as part of the global trachoma mapping project. *Ophthalmic Epidemiol*. 2016;23(sup1):94–99. doi:10.1080/09286586.2016.1250917. PMC5706977 27918232

[23] Duale AB, Ayele NN, Macleod CK, et al. Epidemiology of trachoma and its implications for implementing the “SAFE” strategy in Somali Region, Ethiopia: results of 14 population-based prevalence surveys. *Ophthalmic Epidemiol*. 2018. doi: 10.1080/09286586.2017.1409358. 10.1080/09286586.2017.1409358 PMC6444207 30806549

[24] Negash K, Macleod C, Adamu A, et al Prevalence of trachoma in the Afar Region of Ethiopia: results of seven population-based surveys from the global trachoma mapping project. *Ophthalmic Epidemiol*. 2018. DOI: 10.1080/09286586.2017.1362008. 10.1080/09286586.2017.1362008 PMC6319167 30806550

[25] Central Statistical Agency of Ethiopia *Population and Housing Census Report 2007*. Addis Ababa: Federal Democratic Republic of Ethiopia Population Census Commission; 2008.

